# Accumulation of RNA-dependent protein kinase (PKR) in the nuclei of lung cancer cells mediates radiation resistance

**DOI:** 10.18632/oncotarget.9428

**Published:** 2016-05-18

**Authors:** Chuncheng Hao, Ruping Shao, Uma Raju, Bingliang Fang, Stephen G. Swisher, Apar Pataer

**Affiliations:** ^1^ Departments of Thoracic and Cardiovascular Surgery, The University of Texas MD Anderson Cancer Center, Houston, Texas, USA; ^2^ Current Address: Department of Oncology Radiotherapy, the Cancer Hospital of Harbin Medical University, Harbin, China; ^3^ Department of Experimental Radiation Oncology, The University of Texas MD Anderson Cancer Center, Houston, Texas, USA

**Keywords:** lung cancer, PKR, radiation sensitivity

## Abstract

We have previously demonstrated that radiation induced cell death in PKR (−/−) deficient mouse embryo fibroblasts (MEFs) but not in PKR (+/+) wild type MEFs. Our study indicated that PKR can also be involved in survival pathways following radiation therapy through activation of the AKT survival pathways in these MEFs is mediated in part through PKR. The role of PKR on radiation sensitivity in cancer cells has not been evaluated. In this study, we demonstrated that radiation treatment causes nuclear translocation of PKR in human lung cancer cells. The transduction of lung cancer cells with a dominant negative adenoviral PKR vector blocks nuclear translocation of PKR and leads to the reversal of radiation resistance. Plasmid transduction of lung cancer cells with nuclear targeted wild type PKR vectors also increased radiation resistance. This effect is selectively abrogated by plasmid transduction of dominant negative PKR vectors which restore radiation sensitivity. These findings suggest a novel role for PKR in lung cancer cells as a mediator of radiation resistance possibly through translocation of the protein product to the nucleus.

## INTRODUCTION

RNA-dependent protein kinase (PKR) is an interferon-induced, double-stranded (ds), RNA-activated serine/threonine protein kinase [1, 2]. PKR has a well-established role in anti-viral defense mechanisms, as well as in other cellular functions such as growth control, apoptosis regulation, cell proliferation, signal transduction, and differentiation [3–6]. It has also been demonstrated that PKR can be involved in the cellular response to genotoxic stress [7]. PKR (+/+) wild type mouse embryo fibroblasts (MEFs) are resistant to bulky adduct DNA damage caused by cisplatin, melphalan, and ultraviolet radiation but not to other DNA-damaging agents such as doxorubicin [7]. Other studies have demonstrated that the induction of PKR by pIC or interferon (IFN)-β increases the resistance of Ramos cells (a B lymphoma cell line) to mercury by a mechanism requiring the catalytic activity of PKR [8].

We have demonstrated that PKR can also play a significant role in tumor suppression through the induction of apoptotic pathways [9–12]. On the other hand our study indicate that PKR can also be involved in survival pathways following radiation therapy through activation of the AKT survival pathways in MEFs [13]. The role of PKR in radiation resistance in cancer cells, however, has not previously been demonstrated and the precise mechanism(s) through which PKR confers radioresistance is not known. In this study, we evaluated the role of PKR in radiosensitivity of human lung cancer cells and demonstrated that radiation treatment causes nuclear translocation of PKR in human lung cancer cells. We demonstrated that the transduction of lung cancer cells with adenoviral wt PKR results in radiation resistance. The transduction of lung cancer cells with a dominant negative adenoviral PKR vector blocks nuclear translocation of PKR and leads to the reversal of radiation resistance. Plasmid transduction of lung cancer cells with nuclear targeted wt PKR vectors also results in radiation resistance and this effect is selectively abrogated by plasmid transduction of dominant negative PKR vectors which restore radiation sensitivity. These findings suggest a novel role for PKR in lung cancer cells as a mediator of radiation resistance possibly through translocation of the protein product to the nucleus.

## RESULTS

### Cellular expression and localization of PKR in lung cancer cells following radiation treatment

**Figure 1:**
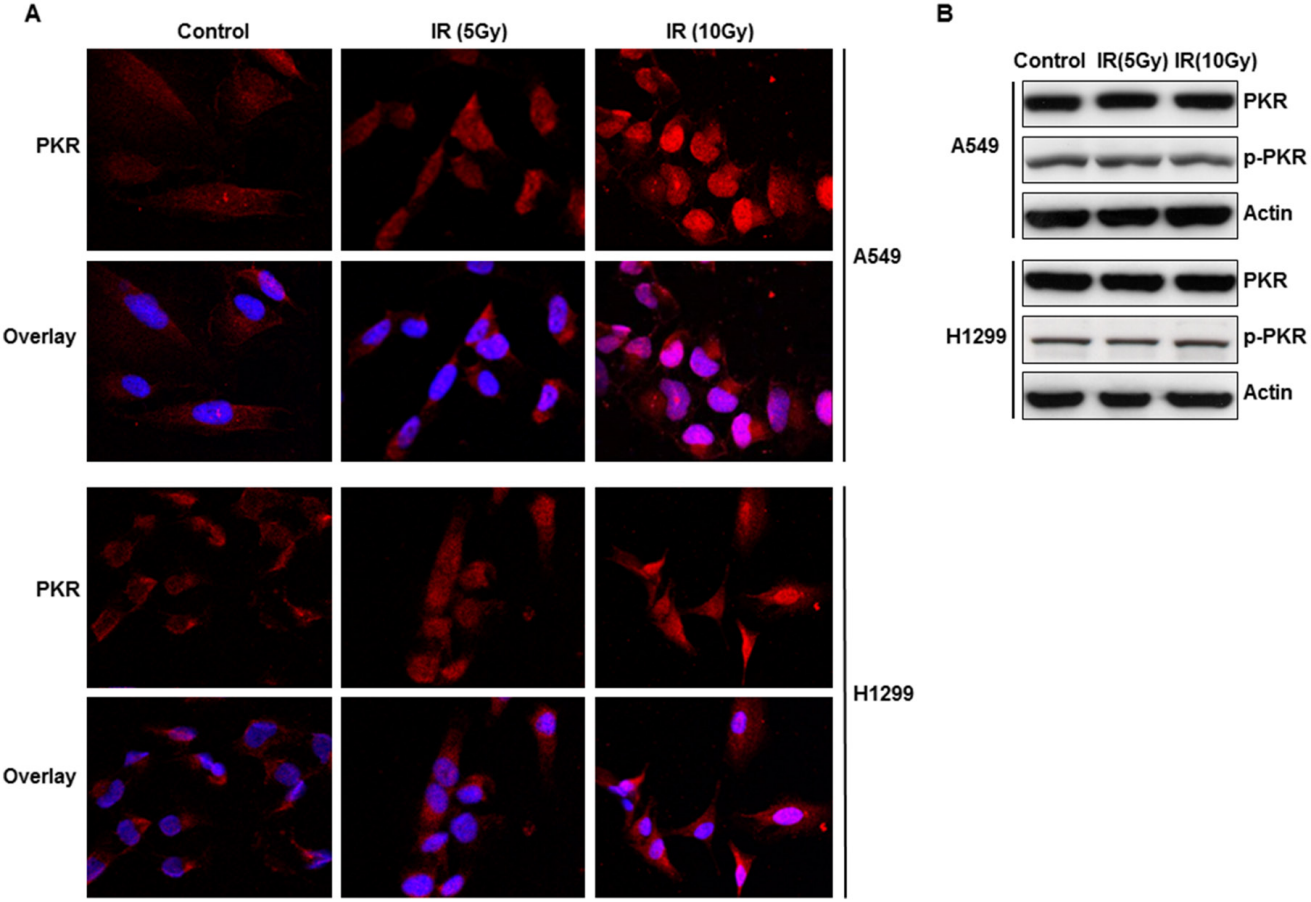
Subcellular localization and expression of PKR in A549 and H1299 cancer cells after radiation treatment **A.** Immunofluorescence microscopy with antibodies against PKR (red) and the nucleus (blue=DAPI staining for DNA in the nucleus) demonstrated accumulation of PKR in the nuclei of A549 and H1299 lung cancer cells after 48 hrs of treatment with radiation. **B.** Western blot analysis of PKR and p-PKR, protein expression in A549 and H1299 lung cancer cells after 48 hrs of radiation treatment. The expression of actin was used as a control.

We investigated the PKR expression and pattern of PKR expression in A549 and H1299 lung cancer cells following radiation treatment by using confocal immunofluorescence analysis and Western blot analysis. We observed that most of the PKR in untreated A549 and H1299 cancer cells was located in the cytosol. However, immunofluorescence analysis revealed that radiation treatment caused translocation of PKR from the cytosol into the nucleus (Figure 1A). Treatment of human lung cancer cells with radiation did not increase the expression of PKR or its phosphorylation (Figure 1B). These results suggest that the accumulation of PKR in the nuclei of these cells may play a role in the development of radiation resistance.

### Adenoviral mutant PKR vector (Ad-PKRΔ6) sensitizes cancer cells to radiation treatment by blocking nuclear translocation of PKR

**Figure 2:**
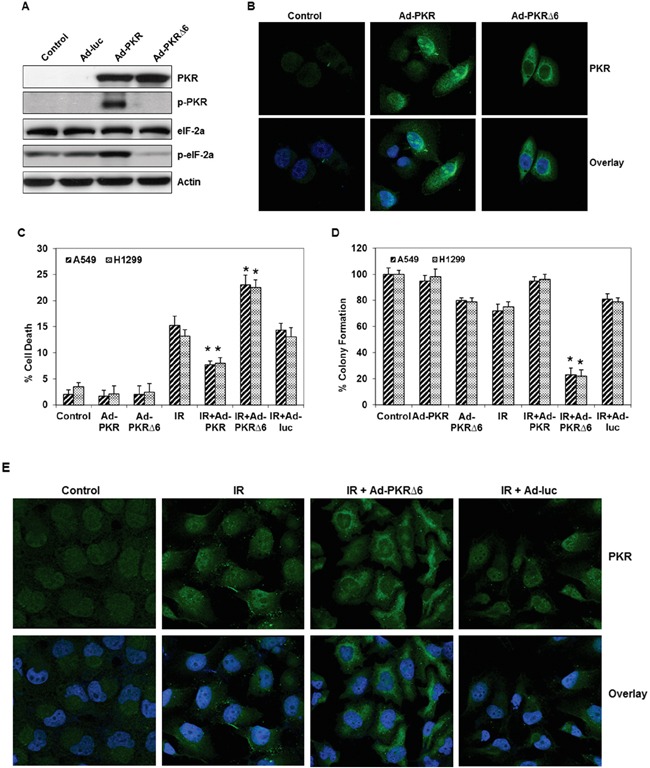
Adenoviral mutant PKR (Ad-PKRΔ6) enhanced radiation-mediated cell death in lung cancer cells **A.** Western blot analysis of PKR, phospho-PKR (p-PKR) and phospho-eIF-2α (p-eIF-2α) protein expression after 48 hrs infected with Ad-PKR or Ad-PKRΔ6 in PKR−/− MEF cells. The expression of actin was used as a control. **B.** Immunofluorescence microscopy with antibodies against PKR (green) and the nucleus (blue=DAPI staining for DNA in the nucleus) demonstrated accumulation of PKR in the cytosol and nuclei of lung cancer cells after 48 hrs of treatment with Ad-PKR. In contrast, Ad-PKRΔ6 treatment increased the level of PKR only in the cytosol of these cancer cells after 48 hrs. **C.** Flow cytometric analysis of apoptosis in adenoviral wild-type (Ad-PKR, 1500 vp) or mutant PKR (Ad-PKRΔ6, 1500 vp)–transfected H1299 and A549 lung cancer cells, with or without radiation treatment. Triplicate experiments were performed for each cell line. **D.** Clonogenic survival assay of A549 and H1299 lung cancer cells treated with adenoviral vector (1500 vp) and radiation (10 Gy). Cells were treated with adenoviral vector and radiation. Treatment with Ad-PKRΔ6 sensitized A549 and H1299 cancer cells to radiation therapy. **E.** Immunofluorescence microscopy with antibodies against PKR (green) and the nucleus (blue=DAPI staining for DNA in the nucleus) demonstrated the accumulation of PKR in the nuclei of A549 lung cancer cells after treatment with radiation. Radiation plus Ad-PKRΔ6 treatment resulted in the accumulation of PKR in the cytosol of these cancer cells after 48 hrs. *, p < 0.05 by Wilcoxon rank-sum test compared with corresponding control (IR, IR+Ad-PKR or IR+Ad-Luc) group.

We next used an adenoviral vector carrying either the wild-type PKR gene or a mutant form (PKRΔ6) to determine how these vectors affect radiation sensitivity in lung cancer cells. The PKRΔ6 products have a deletion of six amino acids (361-366, LFIQME) between catalytic domains IV and V and cannot autophosphorylate or activate substrates [14, 15]. We first evaluated by Western blot analysis the effects of PKR and p-PKR on PKR−/− MEFs transfected with wild-type or mutant PKR via an adenoviral vector. High levels of PKR protein expression were observed in PKR null cells infected by Ad-PKR or Ad-PKRΔ6 (Figure 2A and 2B), whereas no endogenous PKR protein was observed in cells infected with the control construct (Ad-Luc). Cells infected with Ad-PKR or Ad-PKRΔ6 had comparable expression of the PKR protein, but only PKR−/− MEFs infected by Ad-PKR had increased expression of p-PKR (Thr^451^) and p-eiF-2α (Ser^51^). We did not detect increased expression of p-PKR and p-eiF-2α in PKR null MEFs infected with Ad-PKRΔ6 (Figure 2A). We next analyzed the pattern of PKR expression in PKR−/− MEFs after treatment with PBS, Ad-PKR, or Ad-PKRΔ6. Confocal immunofluorescence analysis revealed that PKR levels were markedly higher in both the cytosol and nuclei of PKR−/− MEFs after treatment with Ad-PKR. In contrast, Ad-PKRΔ6 increased the level of PKR in the cytosol only, suggesting that PKR phosphorylation is necessary for translocation into the nucleus (Figure 2B). Taken together, our findings demonstrate that p-PKR may be necessary for development of radiation resistance.

Next, we investigated whether the inhibition of phosphorylated PKR by Ad-PKRΔ6 could enhance radiation-mediated cell death and whether the induction of phosphorylated PKR by Ad-PKR could inhibit radiation-mediated cell death in lung cancer cells. We examined the effects of Ad-PKRΔ6 and Ad-PKR, alone and in combination with radiation (10 Gy) in A549 and H1299 lung cancer cell lines. Flow cytometric analysis showed that Ad-PKRΔ6 as a single agent resulted in cell death rates of 2% in A549 cells and 2.4% in H1299 cells. Radiation (10 Gy) treatments resulted in cell death rates of 15.2% in A549 cells and 13.2% in H1299 cells after 48 hrs. The combination of Ad-PKRΔ6 and radiation resulted in a substantial enhancement of apoptosis in both A549 (23%) and H1299 (22.5%) lung cancer cells (Figure 2C). No such enhancement of apoptotic effects occurred after treatment of the cells with a combination of radiation and Ad-luc. We also found less apoptosis after treatment of the cells with a combination of radiation and Ad-PKR (Figure 2C). To determine the long-term effect of radiation, Ad-PKR or Ad-PKRΔ6 treatment on cell growth, a clonogenic assay was used. As an individual treatment, radiation caused up to a 28% and 25% reduction in clonogenic potential in A549 and H1299, respectively (Figure 2D). Ad-PKRΔ6 treatment caused up to a 20% and 21% reduction in A549 and H1299, respectively (Figure 2D). Treatment with a combination of radiation and Ad-PKRΔ6 resulted in a further decrease (77% and 78% reduction in A549 and H1299, respectively) in clonogenic potential when compared with combination of radiation and Ad-PKR or radiation and Ad-Luc treatment (Figure 2D). We next further investigated whether adenoviral mutant PKR (PKRΔ6) could block translocation of PKR in radiation-treated A549 lung cancer cells. We observed by confocal immunofluorescence analysis that PKR was mostly expressed in the cytosol of untreated cancer cells. We demonstrated that radiation treatment caused the translocation of PKR from the cytosol into the nuclei of the cells. We additionally demonstrated that radiation-induced PKR translocation was blocked by Ad-PKRΔ6 but not by Ad-luc (Figure 2E). Thus, our results suggest that Ad-PKRΔ6 sensitizes cancer cells to radiation treatment by blocking nuclear PKR translocation.

### Nuclear-targeted mutant PKR plasmid sensitizes cancer cells to radiation treatment

**Figure 3:**
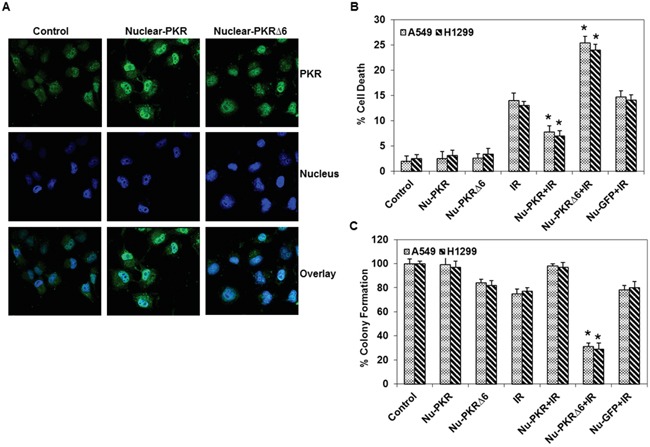
Expression and subcellular localization of PKR in nuclear-targeted wild-type PKR and mutant PKRΔ6-transduced lung cancer cells **A.** Immunofluorescence microscopy with antibodies against PKR (green) and the nucleus (blue=DAPI staining for DNA in the nucleus) demonstrated accumulation of PKR in the nuclei of A549 lung cancer cells after treatment with nuclear-targeted wild-type PKR and mutant PKRΔ6 after 48 hrs. **B.** Flow cytometric analysis of apoptosis in nuclear-targeted GFP and wild-type or mutant PKR-transfected H1299 and A549 lung cancer cells, with or without radiation treatment. Triplicate experiments were performed for each cell line (top). **C.** Clonogenic survival assay of A549 and H1299 lung cancer cells treated with nuclear-targeted plasmids (5 μg) and radiation (10 Gy). Treatment with nuclear-targeted mutant PKRΔ6 sensitized A549 and H1299 cancer cells to radiation therapy. *, p < 0.05 by Wilcoxon rank-sum test compared with corresponding control (IR, IR+Ad-PKR or IR+Ad-Luc) group.

Our findings showed that the accumulation of PKR in the nuclei of lung cancer cells may play a role in the development of resistance. Therefore, we hypothesized that overexpression of PKR in the nuclei of lung cancer cells would make them less sensitive to radiation treatment. We constructed nuclear-targeted wild-type and mutant PKR (PKRΔ6) plasmids (Figure 3A). Figure 3A shows that PKR levels were markedly higher in the nuclei of lung cancer cells after treatment with these plasmids. We next used the plasmids to determine how nuclear-targeted wild-type PKR or PKRΔ6 regulates the response of cancer cells to radiation treatment. We performed flow cytometric analysis on A549 and H1299 lung cancer cell lines after 48 hrs of treatment with nuclear-targeted wild-type PKR or PKRΔ6 plasmids (5 μg), alone and in combination with radiation (10 Gy). We observed that the apoptotic rate was lower in cancer cells transfected with nuclear-targeted wild-type PKR than in control vector (nuclear-targeted GFP)–transfected cancer cells after exposure to radiation (Figure 3B). We further observed that the apoptotic rate was higher in nuclear-targeted PKRΔ6-transfected cancer cells compared with the control vector (nuclear-targeted GFP)–transfected cancer cells after exposure to radiation (Figure 3B). These results strongly indicate that accumulation of PKR in the nuclei of cancer cells plays a role in the development of resistance to radiation treatment. The radiation sensitivity of lung cancer cell was also confirmed by colony formation assay. As shown in Figure 3C, combination of nuclear-targeted PKRΔ6 and radiation decreased clonogenic survival of A549 (69% reduction) and H1299 (71% reduction). The combination of nuclear-targeted PKR and radiation enhanced clonogenic survival of A549 and H1299 (Figure 3C). The results further establish a potential role of PKR in resistance of lung cancer cells toward radiation treatment.

## DISCUSSION

Many studies have postulated that increasing PKR activity has a net tumor suppressive effect through up-regulation of apoptotic pathways [1–3]. Despite these data, the role of PKR as a tumor suppressor is far from clear, with evidence that PKR activation can lead to neoplastic progression in melanoma and colon cancer cells and decreased sensitivity to conventional chemotherapy agents, presumably through up-regulation of pro-survival pathways such as NF-kB [16]. We have demonstrated that radiation induced cell death in a dose and time-dependent manner in PKR (−/−) MEFs but not in PKR (+/+) MEFs [13]. In current study, we further confirmed that PKR plays a role in the development of resistance to radiation treatment in human lung cancer cells. We demonstrated that radiation treatment causes nuclear translocation of PKR in human lung cancer cells. Our results suggest that the accumulation of PKR in the nuclei of these cells may play a role in the development of radiation resistance. We found that combination of adenoviral mutant PKR vector (Ad-PKRΔ6) and radiation decreased clonogenic survival of A549 and H1299 human lung cancer cells. We also demonstrate that adenoviral mutant PKR vector can sensitize lung cancer cells to radiation by blocking the nuclear translocation of PKR. We suggest that nuclear PKR mediates the cell survival pathway and plays an important role in the resistance of cancer cells to radiation. We observed that the apoptotic rate was lower in nuclear-targeted wild-type PKR–transfected cancer cells compared with that in control vector–transfected cancer cells after exposure to radiation. Our additional studies demonstrated that nuclear-targeted mutant PKR (PKRΔ6) can enhance radiation-mediated cell killing in human lung cancer cells. We further observed that combination of nuclear-targeted PKRΔ6 and radiation decreased clonogenic survival of A549 and H1299 human lung cancer cells. Once activated, PKR translocates to the nucleus, but although the cytoplasmic function of PKR is known, its role in the nucleus has yet to be completely defined. It has demonstrated that both NFAR-1 and −2 (nuclear factors associated with dsRNA) are substrates for PKR [17, 18]. Further analysis indicates that the C terminus of NFAR-2 can associate with proteins involved in RNA processing, such as the FUS/TLS (transformed in liposarcomas) and survival motor neuron (SMN) proteins [17]. It has been demonstrated that FUS/TLS is an interacting molecule of the p65 (RelA) subunit of NF-κB [19]. We have demonstrated that PKR can indirectly regulate NF-κB [13]. It is possible; therefore, that PKR-mediated activation of NF-κB may occur through the FUS/TLS pathway. It has also been reported that FUS−/− MEFs have an enhanced radiation sensitivity compared with FUS+/+ MEFs [20]. This raises the possibility that FUS/TLS may be involved in radioresistance in PKR MEFs and cancer cells. It also has been demonstrated that SMN protein facilitates the assembly of stress granules [21]. Stress response is a protective cellular process induced by a variety of environmental stresses, including chemical exposure, heat shock, and ultraviolet irradiation [22]. It has been shown that radiation activates acidic sphingomyelinase and increases the production of ceramide which in turn can regulate protein synthesis by a mechanism(s) involving both PKR and RAX [23]. Ceramide promotes RAX/PKR association, which strongly correlates with PKR activation. The role of ceramide in this mechanism remains to be determined.

In summary, our study demonstrates for the first time that PKR may play a novel role in the radiation resistance of lung cancer cells possibly through translocation of PKR protein to the nucleus. We also suggest that PKR may be a novel target to increase radiation sensitivity in lung cancer cells since the adenoviral transduction of lung cancer cells with a dominant negative PKR vector leads to significantly increased radiation killing following ionizing radiation therapy. Further research is still required to elucidate the mechanisms of nuclear PKR signaling and to determine whether this system is operative not only in human lung cancer cells but also in other cancer cells.

## MATERIALS AND METHODS

### Cell lines and reagents

Human lung cancer A549 and H1299 cell lines were obtained from the American Type Culture Collection (Manassas, VA). PKR +/+ and PKR−/− MEF cells were obtained from Dr. Glen Barber (University of Miami School of Medicine) [9]. MEF cells were maintained in Dulbecco's modified Eagle's medium (DMEM) containing 10% fetal bovine serum, 10 mM glutamine, 100 units/mL penicillin, and 100 μg/mL streptomycin (Life Technologies, Inc., Grand Island, NY) in a 5% CO_2_ atmosphere at 37°C.

### Adenovirus production and plasmid constructs

Construction of the adenoviral luciferase (Ad-luc) vector has been reported previously [15, 24]. The Ad-GT-wild-type PKR (Ad-PKR) and Ad-GT-mutant-type PKR (Ad-PKRΔ6) vectors were constructed by placing wild or mutant PKR cDNA (obtained from Dr. Glen N. Barber, University of Miami School of Medicine) downstream of the GAL4/TATA promoter (GT) to generate the shuttle plasmids pAd/GT-PKR and pAd/GT-PKRΔ6. These plasmids were cotransfected into 293 cells, along with a 35-kb Cla I fragment purified from human adenoviral type 5, to generate the Ad-GT-PKR and Ad-GT-PKRΔ6 vectors. Purified Ad-GT-PKR and Ad-GT-PKRΔ6 were obtained by expanding the virus in the 293 cells, harvesting the supernatant of those cells, and then subjecting the supernatant to ultracentrifugation on a cesium chloride gradient. Virus titers were determined by optical absorbency at A_260_ (1 A_260_ unit=10^12^ viral particle/mL). The transduction efficiencies of adenoviral vectors in A549 and H1299 cancer cell lines were determined by infecting cells with Ad-LacZ and then quantifying the titers needed to transduce at least 70% of the cells. The pCMV/myc/Nuc/GFP plasmid (Invitrogen, Eugene, OR) was digested by Pst I and Not I to obtain pCMV/myc/Nuc/PKR or pCMV/myc/Nuc/PKRΔ6 plasmid by ligation of the digested fragment with the PKR or PKRΔ6 insert. Plasmid and reagent concentrations were optimized to ensure that more than 50% of the cells were transfected in all of the experiments. The relative number of transfected cells was determined by counting the number of green fluorescent protein-positive cells and then assigning this number a value of 1.0 for each experiment. Twenty random fields were counted in each assay.

### Flow cytometry analysis

We measured apoptotic cells by propidium iodide staining and FACS analysis [15]. Cells were harvested; pelleted by centrifugation; resuspended in phosphate-buffered saline (PBS) containing 50 μg/mL propidium iodide, 0.1% Triton X-100, and 0.1% sodium citrate; and subjected to vortexing prior to FACS analysis (Becton-Dickenson FACScan, Mountain View, CA; FL-3 channel).

### Clonogenic assay

Cell survival after treatment with Adenoviral vector and radiation alone or in combination was measured by clonogenic assay [13]. For all experiments, single cells were seeded into 100-mm culture dishes on day 0 and allowed to attach for 24 h at 37°C in 5% FBS medium. For A549 and H1299, 12,000 cells were plated per dish. The cells were then treated with adenoviral vector (2500 vp), radiation (10 Gy), or a combination of adenoviral vector and radiation. Doses of radiation (10 Gy) were administered by using a ^137^Cs source at a dose rate of 3.7 Gy/minutes. Adenoviral vector were washed out 48 h posttreatment and fresh 5% FBS medium was added. After 10 to 14 days, colonies were fixed in 1.0% crystal violet and 0.5% glacial acetic acid in ethanol, and visible colonies containing ~50 or more cells were counted.

### Immunoblot analysis

At 48 or 72 h after transfection, the cell extracts were prepared and immunoblot assays performed as described elsewhere [25–27]. The following antibodies were used: PKR (B-10), PKR (K-17) and beta-actin (Santa Cruz Biotechnology, Santa Cruz, CA); phospho-PKR [pT451] and phospho-eIF-2α [pS51] (BioSource International, Camarillo, CA).

### Cellular localization studies

A549, H1299, and PKR−/− MEF cells (5 × 10^4^ cells/well) were grown on chamber slides until 70% confluence and then treated with PBS, Ad-luc, Ad-PKR, Ad-PKRΔ6, Nu-PKR, Nu-PKRΔ6, or radiation. Fourty-eight hours later, cells were washed with PBS and fixed with 4% paraformaldehyde/PBS for confocal analysis, as previously described [10, 28]. Cells were blocked with 1% normal goat serum for 1 hour and then incubated overnight at a dilution of 1:100 with the primary anti-PKR antibody. To remove the primary antibody, the slides were rinsed with PBS and incubated with a fluorescein isothiocyanate- or rhodamine-conjugated secondary antibody (Invitrogen) for 1 hour. The slides were then mounted with the ProLong Gold antifade reagent containing 4′, 6-diamidino-2-phenylindole (DAPI; Invitrogen) and analyzed under an Olympus FluoView FV500 laser confocal microscope (Olympus America, Melville, NY) after adjustment for background staining.

### Statistical analysis

Data reported in the figures represent the mean of three independent experiments with standard deviations (SD). Differences were considered significant in all experiments at *P* < 0.05 (significantly different from untreated or treated controls).

## Abbreviations

PKR: RNA-dependent protein kinase
dsRNA: double-stranded RNA
MEFs: mouse embryo fibroblasts
eIF-2α: α-subunit of eukaryotic translation initiation factor 2
VP: viral particle.
